# Temperament, bipolar disorder and addictive disorders: Which personalized and integrated approach?

**DOI:** 10.1192/j.eurpsy.2021.57

**Published:** 2021-08-13

**Authors:** L. Orsolini

**Affiliations:** Department Of Clinical Neurosciences/dimsc, Unit of Clinical Psychiatry, School of Medicine, Polytechnic University of Marche, Ancona, Italy

**Keywords:** bipolar disorder, temperament, Affective disorders, Substance Use Disorder

## Abstract

Affective Disorders are on a clinical continuum in which temperaments and other coexisting or emerging mental conditions may cover the role of risk factors or determinants of specific dimensional aspects of Bipolar Disorder. Overall, it is important to better characterize the psychopathological conditions associated to the clinical picture of an affective disorder in order to perform more personalized and integrated approach for the assessment, diagnosis and treatment of individuals with dual disorder.

**Disclosure:**

No significant relationships.

